# Evolution of the metabolic and regulatory networks associated with oxygen availability in two phytopathogenic enterobacteria

**DOI:** 10.1186/1471-2164-13-110

**Published:** 2012-03-22

**Authors:** Lavanya Babujee, Jennifer Apodaca, Venkatesh Balakrishnan, Paul Liss, Patricia J Kiley, Amy O Charkowski, Jeremy D Glasner, Nicole T Perna

**Affiliations:** 1 Biotechnology Center, University of Wisconsin-Madison, WI, USA; 2 Department of Biomolecular Chemistry, University of Wisconsin-Madison, WI, USA; 3 Department of Plant Pathology, University of Wisconsin-Madison, WI, USA; 4 Department of Genetics, University of Wisconsin-Madison, WI, USA

## Abstract

**Background:**

*Dickeya dadantii *and *Pectobacterium atrosepticum *are phytopathogenic enterobacteria capable of facultative anaerobic growth in a wide range of O_2 _concentrations found in plant and natural environments. The transcriptional response to O_2 _remains under-explored for these and other phytopathogenic enterobacteria although it has been well characterized for animal-associated genera including *Escherichia coli *and *Salmonella enterica*. Knowledge of the extent of conservation of the transcriptional response across orthologous genes in more distantly related species is useful to identify rates and patterns of regulon evolution. Evolutionary events such as loss and acquisition of genes by lateral transfer events along each evolutionary branch results in lineage-specific genes, some of which may have been subsequently incorporated into the O_2_-responsive stimulon. Here we present a comparison of transcriptional profiles measured using densely tiled oligonucleotide arrays for two phytopathogens, *Dickeya dadantii *3937 and *Pectobacterium atrosepticum *SCRI1043, grown to mid-log phase in MOPS minimal medium (0.1% glucose) with and without O_2_.

**Results:**

More than 7% of the genes of each phytopathogen are differentially expressed with greater than 3-fold changes under anaerobic conditions. In addition to anaerobic metabolism genes, the O_2 _responsive stimulon includes a variety of virulence and pathogenicity-genes. Few of these genes overlap with orthologous genes in the anaerobic stimulon of *E. coli*. We define these as the conserved core, in which the transcriptional pattern as well as genetic architecture are well preserved. This conserved core includes previously described anaerobic metabolic pathways such as fermentation. Other components of the anaerobic stimulon show variation in genetic content, genome architecture and regulation. Notably formate metabolism, nitrate/nitrite metabolism, and fermentative butanediol production, differ between *E. coli *and the phytopathogens. Surprisingly, the overlap of the anaerobic stimulon between the phytopathogens is also relatively small considering that they are closely related, occupy similar niches and employ similar strategies to cause disease. There are cases of interesting divergences in the pattern of transcription of genes between *Dickeya *and *Pectobacterium *for virulence-associated subsystems including the type VI secretion system (T6SS), suggesting that fine-tuning of the stimulon impacts interaction with plants or competing microbes.

**Conclusions:**

The small number of genes (an even smaller number if we consider operons) comprising the conserved core transcriptional response to O_2 _limitation demonstrates the extent of regulatory divergence prevalent in the Enterobacteriaceae. Our orthology-driven comparative transcriptomics approach indicates that the adaptive response in the eneterobacteria is a result of interaction of core (regulators) and lineage-specific (structural and regulatory) genes. Our subsystems based approach reveals that similar phenotypic outcomes are sometimes achieved by each organism using different genes and regulatory strategies.

## Background

*Dickeya dadantii *and *Pectobacterium atrosepticum *cause soft-rot diseases characterized by maceration of plant tissues through the action of multiple secreted plant cell wall degrading enzymes [1]. *D. dadantii *strain 3937 (*D. dadantii*) was originally isolated from African violet and is better known by its former *name Erwinia chrysanthemi *3937, and *P. atrosepticum *strain SCRI1043 was isolated from potato [2,3], but individual strains and these genera as a whole have broad host range, affecting over 50% of angiosperm plant orders [4]. They are a world-wide problem for economically important crops and ornamental plants [5]. Both *D. dadantii *and *P. atrosepticum *are relatively well-studied model organisms for understanding the molecular biology of soft-rot pathogenesis [6,7]. Like most enterobacteria, *Dickeya *and *Pectobacterium *are facultative anaerobes that are able to grow with or without O_2 _by shifting metabolic strategies from aerobic respiration to anaerobic respiration or fermentation [8]. They experience a wide range of O_2 _concentrations in different plant tissues and natural reservoirs like soil and water [9]. Lack of O_2 _is thought to be one of the factors that can trigger rapid expansion of latent infections leading to devastating post-harvest destruction of entire crops in storage [5].

Apart from a small number of important virulence factors, such as pectinases PelA, D and E [10], little is known about which genes are regulated by O_2 _availability in these two soft-rot pathogens. In contrast, O_2 _-regulated genes have been extensively studied in the model animal-associated enterobacteria *Escherichia coli *and *Salmonella enterica*, where available data includes genome-scale expression profiling of the anaerobic stimulon of wild-type strains as well as mutants of key regulators FNR, ArcA, NarPQ and NarXL [11-16]. Most of these regulators, and many of the known target genes associated with anaerobic metabolism are conserved across the enterobacteria and among more distantly related gamma-proteobacteria [17]. Thus, we expect a conserved core transcriptional response to O_2 _limitation that includes the basic cellular machinery required to generate energy in an anaerobic environment. Yet, some of the O_2_-regulated genes found in *E. coli *are simply not present in other genera, and other genes that may be O_2 _- responsive in the phytopathogens are not shared with animal-associated organisms. A more complete picture of the anaerobic stimulon of plant-pathogenic enterobacteria requires direct experimentation in these organisms.

Here, we characterize the transcript profiles of *P. atrosepticum *and *D. dadantii *grown with and without O_2 _under controlled laboratory conditions. We performed our experiments in defined media to illuminate solely the O_2_-responsive regulatory network. These conditions are not expected to mirror the complex, dynamic, and largely undefined environment of a plant host. Rather, we seek to identify components of the anaerobic stimulon for follow-up experimentation, provide a framework for identification of characteristics of the response to O_2 _in more complex datasets, and investigate the conservation and divergence in O_2_-mediated regulation among the enterobacteria.

## Results and discussion

### A large number of genes are in the O_2_-response stimulon

Using a conditional false-discovery rate (cFDR) of 0.01 (permissive criterion for differential expression), EBarrays [18] detects over 2204 differentially expressed genes in *D. dadantii *(48.5%), and 599 in *P. atrosepticum *(13.4%). Particularly in *D. dadantii *where the extremely good agreement between replicates improves the sensitivity, many of these are genes that show small changes between the aerobic and anaerobic conditions. Requiring the changes (anaerobic/aerobic) be at least 3-fold reduces the numbers to 443 differentially expressed genes in *D. dadantii *(9.8%) and 320 genes in *P. atrosepticum *(7.3%). Thus, a substantial fraction of each genome is involved in the anaerobic stimulon even using our stringent criteria (cFDR = 0.01 and fold change > 3), consistent with published reports for *E. coli *K-12 [12] despite differences in array platforms and analysis methods. The most extreme and conserved transcriptional responses are associated with anaerobic metabolism indicating that these organisms are responding to O_2 _availability.

### Differences in the genetic architecture of the O_2_-responsive stimulon are illuminated by a biological subsystems approach

*Pectobacterium *and *Dickeya *(with *Brenneria*) form a monophyletic clade of phytopathogens distinct from other genera of enterobacteria, like *Escherichia *and *Salmonella *[4], where the transcriptional response to O_2 _has been extensively studied. Nevertheless, all free-living enterobacteria with sequenced genomes share a substantial fraction of ancestral genes that are thought to reflect clonal or vertical descent. Gene losses, duplications, and lateral gene transfers lead to content differences among genomes. Little is known about the extent to which these types of events factor into variation in the response of different enterobacteria to O_2 _availability.

We used OrthoMCL [19] to cluster protein-coding genes from *D. dadantii*, *P. atrosepticum*, and *E. coli*, and used these ortholog groups to compare transcript profiles across organisms. Genes for some of the well-characterized components of the *E. coli *anaerobic energy metabolism architecture are entirely missing from one or both of the phytopathogens. Some functional equivalents within and between organisms are carried out by genes that are not orthologous. For these reasons, we find it useful to approach the comparison from a biological subsystem-oriented perspective that accommodates the complexity of the evolutionary history and functional redundancy, and considers related gene products, such as genes associated with a single molecular complex or biological process [20]. In the following sections, we report our findings from a largely statistical perspective. We discuss the possible biological significance in later sections following a subsystems oriented approach that groups orthologous, paralogous and even analogous genes with functionally related products.

### Transcriptional response to O_2 _limitation for genes orthologous in the two phytopathogens

**Figure 1:**
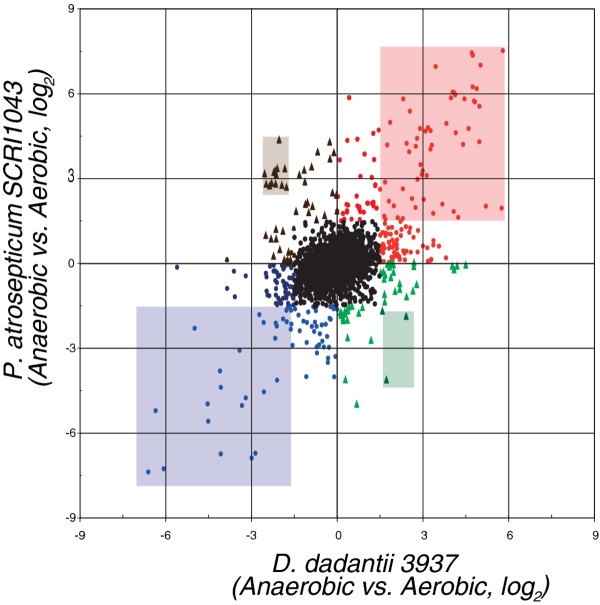
**Scatterplot of fold changes for the 2889 orthologous genes in *D. dadantii *vs P. *atrosepticum***. Fold change values are represented as log ratio of expression in anaerobic vs. aerobic condition. The categories represented are: Statistically significant up-regulation with fold changes greater than 3 (log_2 _> 1.5) in both organisms (orange box), Statistically significant down-regulation with fold changes greater than 3 (log_2 _> 1.5) in both organisms (purple box), Statistically significant expression in both organisms but in opposite directions (up-regulated in *D. dadantii*: green box, down-regulated in *D. dadantii*: brown box) and Equivalently expressed in both organisms (black). At least 81 genes are part of a highly differentially regulated core of genes conserved across *D. dadantii *and *P. atrosepticum *(orange and purple boxes) and this minimum core grows to 222 genes if we allow smaller, but statistically significant differences between aerobic and anaerobic samples (red and blue filled circles). We observe divergent expression patterns in which orthologs are up-regulated > 3- fold in one organism and down-regulated > 3- fold (log_2 _> 1.5) in the other organism for 15 ortholog sets (brown and green boxes), and at least 35 additional ortholog sets show a less extreme, but nevertheless divergent pattern (brown and green filled triangles). Genes that are differentially expressed in one organism only, have not been distinguished in the figure. Given the overall larger number of genes called differentially expressed for *D. dadantii *relative to *P. atrosepticum*, there are many cases where the ortholog in one organism is called differentially expressed (and with fold change > 3), while the ortholog in the other genome is not (966 ortholog groups in *D. dadantii *and 77 different groups in *P. atrosepticum*). In most cases both members of the ortholog group trend in the same direction (766 orthologs) rather than exhibiting divergent expression (277 orthologs).

In total, our OrthoMCL analysis clustered 3110 *D. dadantii *genes and 3094 *P. atrosepticum *genes into groups that contained at least one gene from both phytopathogens. Of these, 2889 groups are simple 1-1 cases and their expression patterns are summarized in Figure 1. Overall, far more of the 1-1 orthologs differentially expressed under aerobic and anaerobic conditions exhibit congruent rather than divergent expression patterns. This observation is not surprising since the two pathogens supposedly share a common ancestor and occupy similar ecological niches. Among genes that are detected as differentially expressed using stringent criteria (cFDR = 0.01 and fold change > 3), 247 *D. dadantii *genes and 196 *P. atrosepticum *genes have predicted orthologs in the other genome (Additional File 1). Of these, 96 show fold changes > 3 for both orthologs. Eighty-one ortholog sets show congruent changes in expression in both organisms; transcripts for 51 are "up-regulated" (transcript abundance is higher under anaerobic conditions) and 30 are "down-regulated" (transcript abundance is lower under anaerobic conditions). Together these represent a minimal conserved transcriptional response shared by both plant pathogens (Table 1). Not surprisingly, the majority of the up-regulated genes encode essential cellular functions under anaerobic conditions. Beside these, 15 ortholog sets change expression in opposite directions in *D. dadantii *and *P. atrosepticum *(Table 2). Interestingly, 12 of the 15 ortholog sets belong to a single biological system, namely type VI secretion system.

**Table 1:** Minimal conserved anaerobic transcriptional response shared by *D. dadantii *3937 and *P. atrosepticum *SCRI1043 and its comparison to *E. coli*.

ASAP Feature ID			Gene Name	Product	Fold change		
*D. dadantii*	*P. atrosepticum*	*E. coli*			*D. dadantii*	*P. atrosepticum*	*E. coli*
**A. Orthologs down-regulated ****≥3-fold in both *****D. dadantii *and *P. atrosepticum***							
ABF-0016541	ABL-0060576	ABE-0009869	*exbB*	membrane spanning protein in TonB-ExbB-ExbD complex	-6.6	-3.5	-3.3
ABF-0020799	ABL-0061445	ABE-0007383	*nrdA*	ribonucleotide reductase of class Ia (aerobic), alpha subunit	-4.3	-4.7	-2.2*
ABF-0020798	ABL-0061444	ABE-0007386	*nrdB*	ribonucleotide reductase of class Ia (aerobic), beta subunit	-3.7	-3.9	-2.0*
ABF-0017172	ABL-0063446	ABE-0009169	*sdaC*	serine transporter	-3.1	-3.2	1.3*
ABF-0020233	ABL-0063426		*sfuA*	iron-binding periplasmic protein	-9.1	-26.9	
ABF-0019943	ABL-0062041	ABE-0003583	*yceI*	secreted protein	-3.1	-5.0	-6.0
ABF-0019942	ABL-0062042	ABE-0003585	*yceJ*	predicted cytochrome b561	-4.4	-4.3	-8.6
ABF-0020068	ABL-0062100	ABE-0005694	*ydiU*	hypothetical protein	-3.4	-4.2	-2.2*
ABF-0015019	ABL-0064500	ABE-0012474	*yigI*	conserved protein	-2.9	-7.4	-3.1
ABF-0019535	ABL-0061414	ABE-0001021	*ykgM*	predicted ribosomal protein	-97.0	-165.4	-1.4*
ABF-0019536	ABL-0061413	ABE-0285027	*ykgO*	predicted ribosomal protein	-66.7	-153.3	
ABF-0017084	ABL-0062748	ABE-0006191	*znuA*	zinc ABC transporter, periplasmic-binding protein ZnuA	-4.3	-17.5	-1.2*
ABF-0017082	ABL-0062750	ABE-0006201	*znuB*	high-affinity zinc transport system membrane protein	-3.3	-2.9	1.4*
ABF-0017083	ABL-0062749	ABE-0006198	*znuC*	high-affinity zinc transport system ATP-binding protein	-3.1	-2.8	-1.1*
ABF-0018178	ABL-0063535			Iron dicitrate-binding protein	-10.6	-8.4	
ABF-0018571	ABL-0064593			putative iron ABC transporter permease protein	-5.9	-23.3	
ABF-0018572	ABL-0064592			putative iron ABC transporter, periplasmic-binding protein	-7.9	-117.8	
ABF-0018573	ABL-0064591			putative iron ABC transporter ATP-binding protein	-7.3	-104.7	
ABF-0018864	ABL-0063083			TonB-dependent ferric achromobactin receptor	-31.6	-4.9	
ABF-0019222	ABL-0064073			putative ABC transporter substrate-binding protein	-16.8	-106.2	
ABF-0019223	ABL-0064074			putative ABC transporter substrate-binding protein	-22.8	-47.8	
ABF-0019568	ABL-0061801			putative transport system permease protein	-10.1	-32.4	
ABF-0019569	ABL-0061802			putative ABC transporter substrate-binding protein	-23.1	-31.3	
ABF-0019570	ABL-0061804			putative ABC transporter substrate-binding protein	-16.7	-20.8	
ABF-0019572	ABL-0061805			putative ABC transporter substrate-binding protein	-17.0	-13.9	
ABF-0020094	ABL-0060663			ABC-type transporter, periplasmic component	-4.5	-6.3	
ABF-0020095	ABL-0060662			ABC transporter, permease protein	-5.9	-4.3	
ABF-0020096	ABL-0060661			ABC transporter, permease protein	-3.0	-4.4	
ABF-0020097	ABL-0060660			ABC transporter ATP-binding protein	-3.4	-3.2	
ABF-0046525	ABL-0064075			ABC transporter substrate-binding protein	-81.6	-37.0	
**B. Orthologs up-regulated ****≥3-fold in both *****D. dadantii *****and *****P. atrosepticum***							
ABF-0020642	ABL-0062590	ABE-0004164	*adhE*	iron-dependent alcohol dehydrogenase	16.8	3.6	3.9
ABF-0018570	ABL-0064258	ABE-0002090	*ahpC*	alkyl hydroperoxide reductase, C22 subunit	2.8	6.1	-2.2*
ABF-0019339	ABL-0060528		*budC*	2,3-butanediol dehydrogenase	36.8	4.1	
ABF-0018628	ABL-0061786	ABE-0013503	*dcuB*	C4-dicarboxylate transporter DcuB	16.4	66.3	8.5
ABF-0018914	ABL-0063040	ABE-0002776	*dps*	Fe-binding and storage protein	3.2	2.9	-2.5*
ABF-0019603	ABL-0062860	ABE-0003073	*focA*	formate transporter	8.1	4.8	3.1
ABF-0017842	ABL-0064288	ABE-0013604	*frdA*	fumarate reductase (anaerobic) NAD/flavoprotein subunit	7.5	11.2	3.5
ABF-0017841	ABL-0064289	ABE-0013602	*frdB*	fumarate reductase (anaerobic), Fe-S subunit	8.8	8.6	5.3
ABF-0017839	ABL-0064290	ABE-0013598	*frdC*	fumarate reductase (anaerobic), membrane anchor subunit	7.9	9.6	3.7
ABF-0017837	ABL-0064291	ABE-0013595	*frdD*	fumarate reductase (anaerobic), membrane anchor subunit	7.0	7.8	4.5
ABF-0019825	ABL-0062949	ABE-0002893	*grxA*	glutaredoxin 1,coenzyme for ribonucleotide reductase	4.3	3.1	1.0*
ABF-0017078	ABL-0061498		*hoxN*	high-affinity nickel transport protein	6.7	17.6	
ABF-0017349	ABL-0061473	ABE-0009830	*hybB*	predicted hydrogenase 2 cytochrome b type component	5.4	18.8	2.6*
ABF-0017353	ABL-0061476	ABE-0009824	*hybE*	hydrogenase 2-specific chaperone	5.7	15.3	2.1*
ABF-0017346	ABL-0061471	ABE-0009834	*hybO*	hydrogenase 2, small subunit	3.4	18.1	14.6
ABF-0015747	ABL-0061483	ABE-0008931	*hycI*	protease involved in processing C-terminal end of HycE	9.6	25.8	1.4*
ABF-0015752	ABL-0061495	ABE-0008919	*hydN*	formate dehydrogenase-H, ferredoxin subunit	55.3	183.5	1.7*
ABF-0015735	ABL-0061493	2 orthologs	*hyfA*	hydrogenase 4, 4Fe-4S subunit	32.2	128.0	MO
ABF-0015736	ABL-0061492	2 orthologs	*hyfB*	hydrogenase 4, membrane subunit	26.7	163.1	MO
ABF-0015737	ABL-0061491	2 orthologs	*hyfC*	hydrogenase 4, membrane subunit	10.9	123.6	MO
ABF-0015738	ABL-0061490	ABE-0008185	*hyfD*	hydrogenase 4, membrane subunit	26.2	173.6	-1.7*
ABF-0015739	ABL-0061489	ABE-0008188	*hyfE*	hydrogenase 4, membrane subunit	29.4	72.0	1.5*
ABF-0015740	ABL-0061488	ABE-0008191	*hyfF*	hydrogenase 4, membrane subunit	15.8	57.3	2.7*
ABF-0015741	ABL-0061487	2 orthologs	*hyfG*	hydrogenase 4, subunit	26.7	75.1	MO
ABF-0015742	ABL-0061486	ABE-0008942	*hyfH*	hydrogenase 4, Fe-S subunit	17.0	64.9	1.9*
ABF-0015744	ABL-0061485	2 orthologs	*hyfI*	hydrogenase 4, Fe-S subunit	17.5	62.2	MO
ABF-0015745	ABL-0061484	2 orthologs	*hyfJ*	predicted processing element hydrogenase 4	31.3	46.9	MO
ABF-0017358	ABL-0061480	ABE-0008960	*hypB*	GTP hydrolase involved in nickel liganding into hydrogenases	5.8	41.4	2.3*
ABF-0020729	ABL-0061479	ABE-0008962	*hypC*	[NiFe] hydrogenase metallocenter assembly protein HybG	21.1	18.4	3.8
ABF-0017360	ABL-0061478	ABE-0008965	*hypD*	protein required for maturation of hydrogenases	3.6	31.6	2.4*
ABF-0047122	ABL-0062652	ABE-0006058	*manZ*	mannose-specific enzyme IID component of PTS	3.9	4.1	-2.1*
ABF-0016556	ABL-0060593	ABE-0013865	*nrdD*	anaerobic ribonucleoside-triphosphate reductase	17.8	24.4	4.4
ABF-0016554	ABL-0060592	ABE-0013860	*nrdG*	anaerobic ribonucleotide reductase activating protein	4.9	6.2	4.5
ABF-0017768	ABL-0062712	ABE-0003800	*pepT*	peptidase T	31.3	19.7	3.6
ABF-0019604	ABL-0062861	2 orthologs	*pflB*	pyruvate formate lyase I	10.3	3.4	MO
ABF-0174126	ABL-0064936	ABE-0003227	*rmf*	ribosome modulation factor	8.3	3.2	1.9*
ABF-0015967	ABL-0063825	ABE-0008501	*trxC*	thioredoxin 2	7.0	21.3	-3.8
ABF-0016966	ABL-0061596	ABE-0002386	*ybfA*	predicted protein	6.7	3.9	2.2*
ABF-0019390	ABL-0062816	ABE-0003125	*ycbJ*	conserved protein	6.1	4.9	3.3
ABF-0018000	ABL-0062073	ABE-0003740	*ycfP*	conserved protein	3.1	3.2	1.7*
ABF-0020593	ABL-0063322	ABE-0007565	*yfbS*	predicted transporter	3.5	4.6	1.0*
ABF-0020590	ABL-0063324	ABE-0007571	*yfbU*	conserved protein	3.2	7.3	1.4*
ABF-0020347	ABL-0063577	ABE-0008489	*yfiD*	pyruvate formate lyase subunit	18.8	3.1	5.9
ABF-0018102	ABL-0060954	ABE-0010380	*yhbU*	predicted peptidase (collagenase-like)	8.5	25.5	5.9
ABF-0018103	ABL-0060953	ABE-0010382	*yhbV*	predicted protease	9.9	18.0	3.7
ABF-0015647	ABL-0060483	ABE-0010667	*yhdH*	predicted oxidoreductase, Zn-dependent and NAD(P)-binding	4.9	3.0	1.8*
ABF-0020757	ABL-0062521	ABE-0005319	*ynfK*	predicted dethiobiotin synthetase	9.6	16.3	5.3
ABF-0017163	ABL-0063441			putative membrane protein	7.8	8.8	
ABF-0018208	ABL-0060635			hypothetical protein	14.1	30.7	
ABF-0018787	ABL-0063809			lactoylglutathione lyase-like lyase	53.8	3.8	
ABF-0019032	ABL-0061661	ABE-000492*1*		formate dehydrogenase, cytochrome B556 subunit	24.3	27.1	1.5*

This list of orthologous phytopathogen genes which have a 1-1 relationship according to OrthoMCL are differentially expressed with a 3- fold or more change in gene expression for both orthologs. Where there is a single *E. coli *ortholog in the group, the fold change is included, and, statistically insignificant values are marked with an asterisk. Several groups have multiple orthologs in *E. coli*, (indicated by MO) and their expression values are not included. Orthologs showing congruent expression pattern with fold changes > 3 across all three organisms have gene names in bold. The gene names and products were selected from among the three organisms to favor the most correct or most potentially informative and edited slightly to unify the format.

**Table 2:** Comparison of divergent and differentially expressed genes in the phytopathogens to *E. coli*.

ASAP Feature ID			Gene Name	Product	Fold change		
*D. dadantii*	*P. atrosepticum*	*E. coli*			*D. dadantii*	*P. atrosepticum*	*E. coli*
ABF-0014955	ABL-0064548	ABE-0003424	*putA*	Proline dehydrogenase	5.3	-3.7	1*
ABF-0015852	ABL-0063738	ABE-0002201		putative lipoprotein	-3.4	6.3	1.2*
ABF-0015853	ABL-0063739			putative membrane protein	-5.1	6.5	
ABF-0015854	ABL-0063740			IcmF-related protein	-4.9	7	
ABF-0015858	ABL-0063744			putative chaperone	-4.3	10	
ABF-0015859	ABL-0063745			putative membrane protein	-5.8	8.8	
ABF-0015860	ABL-0063746			hypothetical protein	-3.6	9.9	
ABF-0015861	ABL-0063747			putative lipoprotein	-4.4	8.6	
ABF-0015862	ABL-0063748			hypothetical protein	-4.5	9.4	
ABF-0015864	ABL-0063749			hypothetical protein	-4.4	6.8	
ABF-0015865	ABL-0063750			hypothetical protein	-5.6	6.8	
ABF-0015866	ABL-0063751			hypothetical protein	-4.6	9.1	
ABF-0015868	ABL-0063752			hypothetical protein	-3.8	6.6	
ABF-0018340	ABL-0061543	ABE-0002155	*lipA*	lipoate synthase	3	-3.3	-1.4*
ABF-0018771	ABL-0062484		*cybC*	soluble cytochrome b562	3.3	-17.6	

This list of orthologous phytopathogen genes have a 1-1 relationship according to OrthoMCL and are differentially expressed with a 3 fold or more change in gene expression for both orthologs, but in opposite directions. Where there is a single *E. coli *ortholog in the group, the fold change is included, and, statistically insignificant values are marked with an asterisk. The gene names and products were selected from among the three organisms to favor the most correct or most potentially informative and edited slightly to unify the format.

If we include simple ortholog groups where both orthologs are detected as differentially expressed (cFDR = 0.01), but one or the other, or even both have fold changes < 3, then we identify 222 ortholog groups in total that are differentially expressed in a congruent direction, and 51 that are differentially expressed in divergent directions across the two phytopathogens (Additional File 2). This provides a more generous estimate of the conserved core and divergently expressed members of the stimulon. This more permissive congruent set includes operons associated with anaerobiosis in other organisms and closer inspection shows that in these cases, some genes do meet our stringent criteria. This observation provides evidence that our more permissive congruent set includes real members of the anaerobic stimulon. Below, where we detail the genes and biological processes that are implicated in the transcriptional response to O_2_, we guide inclusion by the stringent set (96 genes), but do not limit discussion to genes meeting the stringent significance criteria.

### Comparison of the expression patterns for orthologs shared by *D. dadantii*, *P. atrosepticum *and *E. coli*

*D. dadantii *and *P. atrosepticum *are more closely related to each other than to *E. coli*. Our OrthoMCL analysis identified 2231 groups that include at least one gene from each of the three organisms (totaling 2309 *E. coli *genes, 2283 *D. dadantii *genes, and 2263 *P. atrosepticum *genes). Of these, 2124 ortholog groups include a single gene from each organism.1124 of the 2124 groups have a differentially expressed gene (permissive criteria) in at least one of the three organisms, and 261 groups have at least one gene that shows fold change > 3 (114 genes in *P. atrosepticum*, 111 in *E. coli*, and 153 in *D. dadantii*, see Additional File 3). Only 20 ortholog groups contain genes that show a congruent expression pattern with fold change greater than 3 for all three orthologs (Table 1, bold gene names), suggesting that the conserved response to O_2 _is small in terms of the number of genes involved, or that the conserved response lies in orthologs with smaller magnitude changes. This set is mainly comprised of genes known to function in cellular metabolism under anaerobic conditions namely, *frdABCD, dcuB, adhE, hypC, focA, hybO, yfiD, nrdG, nrdD*, beside others such as the collagenase encoding genes *yhbUV*, a peptidase coding gene *pepT*, and two other genes *ynfK *and *ycbJ *which are all up-regulated. The stringent congruent set also includes four down-regulated genes. These are *exbB *that encodes a component of the TonB-exbBD complex, *yceI *that encodes a cytochrome b561 and two other genes (*yceJ, yigI*), which encode uncharacterized proteins.

Relaxing the analysis stringency to consider all 1-1-1 orthologs that are differentially expressed (no fold change threshold) in all three organisms results in only a small increase to 39 ortholog groups with congruent expression pattern (see Additional File 2, bold gene names). This suggests that the magnitude of the response is not the primary reason the conserved stimulon is so small. Even our most permissive analysis suggests that there are more genes in 1-1 ortholog groups that are differentially expressed in a subset of the organisms than congruent across all three.

### Transcriptional response to O_2 _limitation for genes orthologous in the two phytopathogens and not shared with *E. Coli*

*D. dadantii *and *P. atrosepticum *share lifestyle characteristics, including a plant-host environment, not common to *E. coli*. They are also more closely related to each other and have acquired genes by lateral transfer events along the shared branch since their divergence from *E. coli*. We examined the set of orthologs shared by the two phytopathogens, but absent from *E. coli *to examine which if any of these lineage-specific genes are O_2_-responsive. A total of 780 OrthoMCL groups include genes from both phytopathogens and none from *E. coli*, and 716 of these are simple 1-1 ortholog groups. Only 22 genes without *E. coli *orthologs are differentially expressed with fold changes greater than 3 in both *D. dadantii *and *P. atrosepticum *(see Table 1). Of these 5 are up-regulated and encode butanediol dehydrogenase, lactoylglutathione lyase, a nickel transporter (different from the *E. coli nikABCDE *nickel transport system), a putative membrane protein and a hypothetical protein. All of the 17 down-regulated genes encode proteins that constitute transport systems, and many of them likely transport iron. These are discussed in greater detail in later sections.

### Transcriptional response of genes shared with *E. Coli *and only one of the phytopathogens

Forty *P. atrosepticum *genes shared with *E. coli *but not with *D. dadantii *are O_2 _responsive in *P. atrosepticum *in our experiments, and for 26 of them transcript levels change more than 3-fold between the conditions. Similarly, 70 *D. dadantii *genes that have orthologs in *E. coli *but not in *P. atrosepticum *are differentially expressed in *D. dadantii*, of which 18 show fold changes > 3 (see Additional File 4).

### Each phytopathogen shows a distinct response to O_2 _limitation - differential expression of genes without orthologs in the other organism

*D. dadantii *and *P. atrosepticum *each encode a substantial number of genes that are not predicted to have orthologs in the other phytopathogen or in *E. coli*. There are 1267 *D. dadantii *protein-coding genes in the ASAP database that were not found in OrthoMCL groups. Of these, 501 are differentially expressed and 142 show fold changes greater than 3. These 142 genes include several which were previously implicated in virulence or growth and survival in plant hosts (Additional File 5), and other recognizable biological processes, but 57 genes encode proteins of unknown function underscoring our incomplete understanding of the response to O_2 _limitation. In *P. atrosepticum*, there are 1130 ungrouped protein-coding genes, of which 144 are differentially expressed and 73 show fold changes greater than 3. The 73 genes include the coronofacic acid synthesis genes all of which are > 3- fold up-regulated, three genes that encode putative oxidoreductases, at least ten genes that encode putative exported proteins, and 13 genes of unknown function (Additional File 6).

We attribute the larger number of unique O_2_-regulated genes from *D. dadantii *to the smaller variance among replicates. But even using the numbers from *P. atrosepticum*, the number of genes (144 and 73) in the organism-specific transcriptional response is comparable to the number of genes (81) in the conserved transcriptional response, or greatly exceeds it, if we limit the core to the 20 differentially expressed genes with fold changes greater than 3 that are shared across all three organisms.

### The anaerobic stimulon

**Figure 2:**
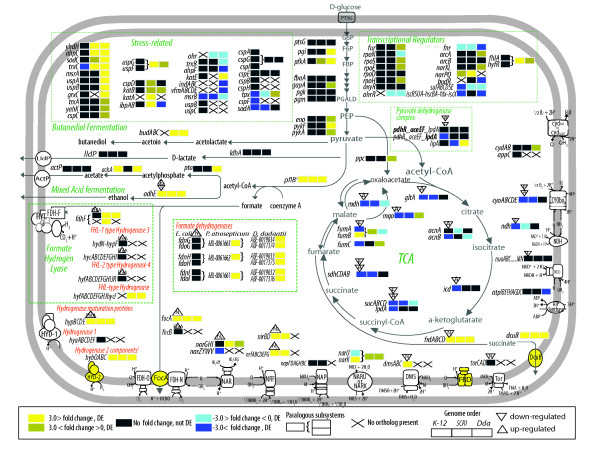
**Metabolic overview of conserved pathways in *E. coli*, *P. atrosepticum *and *D. dadantii***. Changes in gene expression under anaerobic conditions are shown for all three organisms and are represented by different colors. Fold change patterns and genome order are as follows: *E. coli, P. atrosepticum and D. dadantii *(see key within the figure). Each orthologous group of genes is represented by three blocks colored by fold change (dark blue: down-regulated, fold change > 3; light blue: down-regulated, fold change < 3; bright yellow: up-regulated, fold change > 3; dirty yellow: up-regulated, fold change < 3; black: no change in expression, X: ortholog absent in that organism). Fold change values for poly-cistronic operons are averaged across genes. The transcriptional regulators FNR, ArcA, NarP, NarL and FhlA, for which there are known targets, are denoted in the figure based on their mode of regulation: (▲) up regulated (▼) down-regulated. Several components in this figure, such as fermentation and respiratory chains are adapted from Unden and Dunnwald and Sawers et al. 2004 [34,46]. A more detailed diagram of the genomic structure for formate hydrogen lyase complex and accompanying hydrogenases (HYD 1-4) is shown in Figure 3.

The enterobacteria have considerable flexibility, both regulatory and enzymatically, in adapting their metabolism to changing environments. Orthologous genes that demonstrate a response to O_2 _limitation, many of which have 3-fold or more differences in gene expression, are presented according to biological subsystems. The anaerobic growth conditions used in our experiments have limited amounts of alternative electron acceptors (i.e.-nitrate) and are expected to favor fermentation as opposed to anaerobic respiration. Data in the following sections detail the similarities and differences in gene expression patterns of metabolic subsystems conserved across all three organisms (Figure 2) as well as those within and between phytopathogen species(Additional File 7). Additionally, in examining the genomes of these species, we have found many genes associated with the anaerobic stimulon have also undergone considerable changes in genomic architecture. These changes may have an influence on transcriptional differences across each species. These data also demonstrate how the anaerobic stimulon has evolved to include interactions not specifically associated with anaerobic metabolism.

### Global transcriptional regulators associated with anaerobiosis

In *E. coli*, the key O_2 _responsive transcriptional regulators include FNR, ArcAB, NarXL, and NarPQ. FNR is an iron-sulfur cluster-containing protein that dimerizes in the absence of O_2_, and can act as either a transcriptional activator or repressor [21]. The amino acid sequence of FNR from the two phytopathogens is 97.6% identical to the *E. coli *protein with no differences in important functional domains. Surprisingly, *fnr *is more down regulated in *D. dadantii *under anaerobic conditions than either *P*. *atroscepticum *or *E. coli*. If this decreased expression is mediated by FNR as it is in *E. coli*, it suggests that FNR represses its own synthesis to a much greater extent in *D. dadantii *than in *E. coli*.

ArcB is the sensor kinase of the two-component regulatory system, ArcAB, which detects signals emanating from the aerobic respiratory chain, such as the oxidation state of ubiquinones [22]. Under anaerobic conditions ArcB phosphorylates ArcA, activating its site-specific DNA binding activity, which primarily represses genes required for aerobic metabolism [23]. *D. dadantii arcB *contains a nonsense mutation at codon 383, a change that is predicted to interfere with the multi-step phosphorelay transfer to ArcA [24]. It is possible that ArcA is partnered with a different sensor kinase in *D. dadantii*, or that this organism does not have a functional ArcA-mediated regulatory system. Curiously, *arcA *is up-regulated in *D. dadantii*, while *arcA *and *arcB *transcript levels are unaffected in *P. atrosepticum *and *E. coli*.

NarPQ and NarXL are paralogous two-component regulatory systems associated with nitrate/nitrite regulation under anaerobic conditions in *E. coli*. *P. atrosepticum *encodes NarXL and NarPQ of which the latter is missing in *D. dadantii*. The sensor kinase NarX responds to higher concentrations of nitrate than its paralog NarQ, which also responds to nitrite and aeration [25,26]. While each sensor kinase can phosphorylate both response regulators, dephosphorylation to the inactive state for NarP is restricted to the cognate partner [27,28]. Both response regulators activate genes associated with nitrate and nitrite catabolism and repress genes involved in other anaerobic respiratory and fermentative pathways. In *P. atrosepticum*, *narPQ *is up-regulated, while its ortholog in *E. coli*, although not detected as differentially expressed in Kang et al., is up-regulated less than 3- fold.

### TCA cycle and glycolysis

Many genes associated with the central metabolic enzymes of glycolysis and the aerobic TCA cycle, have simple 1-1-1 orthologous relationships. While the transcription patterns are largely conserved, however, most are congruent between *E. coli *and *P. atrosepticum *but different in *D. dadantii *(Figure 2). For example, all four genes of the TCA cycle enzyme succinate dehydrogenase (*sdhCDAB*) are > 3-fold down-regulated in *E. coli *and *P. atrosepticum *but in *D. dadantii *only *sdhD *is down-regulated. These differences may be related to the *arcB *mutation found in *D. dadantii*, which may prevent the anaerobic repression of ArcA regulated genes. In genes that encode isozymes, such as aconitase, all three organisms have at least one isozyme with a conserved response. The fumarase paralogs (*fumA*, *fumB*) are the only TCA cycle genes that do not share simple orthology relationships according to OrthoMCL, although the *fumC *isozymes do. Nevertheless, there is some level of anaerobically induced expression in one of the isozymes across all three organisms (Figure 2).

### Fermentation

Conserved genes that participate in fermentation include those associated with the reduction of pyruvate to lactate (*ldhA*) or the non-oxidative conversion to acetyl-coenzyme-A and formate by pyruvate formate lyase (PFL, *pflB*), and the subsequent conversion to acetate and ethanol (*pta*, *ackA *and *adhE*). In *E. coli*, fermentative lactate dehydrogenase (*ldhA*) is induced under low pH [29]; however under our growth conditions and in the Kang et al. data, no significant changes in gene expression were detected in any organism. The remaining genes *pflB *and *adhE *are up-regulated in all three, however, only *pta *is upregulated in *E. coli and D. dadantii *and *ackA *in *P. atrosepticum *(see Figure 2).

**Figure 3:**
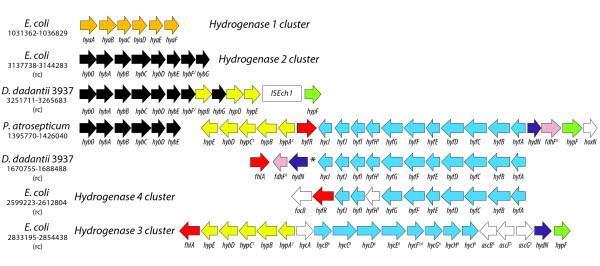
**Genomic architecture of hydrogenase gene clusters from *E. coli*, *D. dadantii *and *P. atrosepticum***. Gene order and orientation of hydrogenase gene clusters from all three organisms are illustrated, including 4 clusters from *E. coli*, 2 from *D. dadantii *3937 and 1 from *P. atrosepticum *SCRI1043. Direction (forward or reverse complement indicated by (rc)) was selected to maximize collinearity with the single hydrogenase cluster from *P. atrosepticum*. Colors are indicative of OrthoMCL grouping unless otherwise indicated by footnotes such that each color marks the genes associated with *E. coli *clusters and members of orthologous groups from *D. dadantii *3937 and *P. atrosepticum *SCRI1043 labeled with the same name. White genes are singletons with no orthologs in the other two organisms. 1. *hypC *has an ortholog in *D. dadantii *3937 that is located elsewhere, 2. *hybF *and *hypA *are grouped by OrthoMCL, 3. *hycF *(*E. coli*) and *hyfH *(*D. dadantii *3937 and *P. atrosepticum *SCRI1043) are grouped by OrthoMCL, but *E. coli *hyfH is not part of the cluster (singleton), 4. *fdhF *has an ortholog in *E. coli *that is located elsewhere, 5. *ascBF *and *ascG *have orthologs in *P. atrosepticum *SCRI1043 that are located elsewhere, 6. OrthoMCL groups most members of the *E. coli *hydrogenase 3 and 4 systems.

There is remarkable genomic and transcriptional variation across all three organisms in the genes associated with formate metabolism. In *E. coli*, formate, in the absence of a terminal electron acceptor, is further dismutated to CO_2 _and H_2 _via the formate hydrogen lyase complex (FHL), a multienzyme complex that includes a cytoplasmic formate dehydrogenase (FDH-H, *fdhF*), a hydrogenase (Hyd-3, encoded by the *hyc *operon) and other components. In *E. coli*, the FHL type hydrogenase groups with genes of the hydrogenase 3 and hydrogenase 4 (*hyf) *systems. Differences in the structure and content of the hydrogenases in the phytopathogens are shown in Figure 3. *D. dadantii *encodes a similar complement of hydrogenases to *P. atrosepticum*, but they are divided into two distinct loci. Expression of the genes encoding hydrogenase 3 in *E. coli*, and the single FHL type hydrogenases in both *D. dadantii *and *P. atrosepticum *are up-regulated.

Expression of *fdhF (*including a paralog of *fdhF *(ABL-0061761) in *P. atrosepticum) *is up-regulated in both phytopathogens, but remains unaffected in *E. coli *where FHL expression is known to be dependent on formate and an acidic pH under fermentative conditions (Figure 2). FHL is regulated by the formate-dependent regulator (*fhlA*) and sigma-54 [30,31], but are not differentially expressed in *E. coli*. The *E. coli *FhlA regulon includes many components (for example, hydrogenases 3, 4) missing in the phytopathogens, and is an obvious example of regulatory divergence where the network is not only smaller in the phytopathogen lineage, but is transcriptionally divergent (Figure 2). Additionally, OrthoMCL groups *flhA *with the *E. coli *regulator of hydrogenase 4 (*hyfR)*, together with single genes, which are differentially expressed, in *D. dadantii *and *P. atrosepticum*.

Two additional *E. coli *formate dehydrogenase and hydrogenase isozymes linked to respiration are discussed in the following section. Unlike the phytopathogens, *E. coli *formate dehydrogenases are the only proteins in *E. coli *that require selenocysteine for assembly and maturation [32-34]. The specific requirement for selenocysteine in the oxidation of formate has been shown for formate dehydrogenase-H (*fdhF*) where replacement with a cysteine residue resulted in a 20-fold less active protein than the wild-type [32,33]. The phytopathogens are missing genes for tRNASec (*selC*), selenocysteine synthase, the specialized translation elongation factor and the selenophosphate synthase (*selD*). The phytopathogens encode cysteine residues into each of the formate dehydrogenases raising the question of whether they have reduced specific activity.

Both *D. dadantii *and *P. atrosepticum *have a *budAB *operon (encoding alpha-acetolactate decarboxylase and acetolactate synthase) adjacent to a divergently transcribed *budR*-like gene encoding a LysR family transcriptional regulator and *budC *(encoding 2, 3-butanediol dehydrogenase). In our experiments *budC *is up-regulated in both phytopathogens. The *bud *genes enable fermentative butanediol production, a feature that limits the channeling of pyruvate to acid producing pathways and thus counteracts the lethal effects of acidification [35]. It has recently been shown that during soft-rot infection the *bud *genes play the essential role of increasing the pH of the plant apoplast to facilitate activity of pectate lyases [36]. The pathway is not present in *Escherichia *or *Salmonella *but is present in other members of the enterobacteria such as *Serratia, Enterobacter, Erwinia*, and *Klebsiella *[37]. In these organisms, butanediol is involved in interactions among plant, animal and insect hosts by acting as a signaling molecule. The mechanism of insect-attraction has been described [38]. It has been shown to produce an anti-inflammatory response in endotoxin-induced lung injury in rats [39,40].

### Aerobic and anaerobic respiration

In the absence of O_2_, *E. coli *is able to reduce a variety of alternate electron acceptors, including fumarate, dimethyl sulfoxide (DMSO), trimethylamine N-oxide (TMAO), nitrate and nitrite [41], to conserve energy through anaerobic respiration using electrons from a variety of donors. The ability to respire fumarate, nitrate and nitrite anaerobically has been reported for *D. dadantii *and *P. atrosepticum *[42] and a subset of the pathways are conserved with *E. coli*, for example, fumarate reductase (*frdABCD*) and nitrate reductase (*narGHI*). Other nitrate/nitrite reductases, which have a complex evolutionary history, are discussed in more detail below. There are no phytopathogen orthologs of the *E. coli *DMSO reductase genes (*dmsABC*), the two TMAO reductases (*torCAD *and *torYZ*) and their associated regulators (*torR*, *torS *and *torT*).

*E. coli *has three nitrate reductases and two nitrite reductases, which are part of the NarL and NarP regulons (see Figure 2). These include two membrane-bound proton-translocating nitrate reductases, (*narGHJI *and *narZYWV *operons), the periplasmic nitrate reductase (*napFDAGHBC *operon), a formate-dependent respiratory nitrite reductase (*nrfABCDEFG*) and the NADH-dependent nitrite reductase (*nirBDC*). The *E. coli narGHJI *operon has orthologs in both phytopathogens, but *narZYWV *does not. The *narGHJI *operon is up-regulated in both *D. dadantii *and *P. atrosepticum*, but is not differentially expressed in *E. coli*. However, *narGHI *in *E. coli *is known to be induced by FNR during anaerobic growth and further induced by NarL [43,44]. Although there are predicted binding sites for NarL and FNR in the regulatory region of *narG *in the phytopathogens, the sequence upstream of the conserved FNR binding site, 53 bp from the transcriptional start, has diverged.

**Figure 4:**
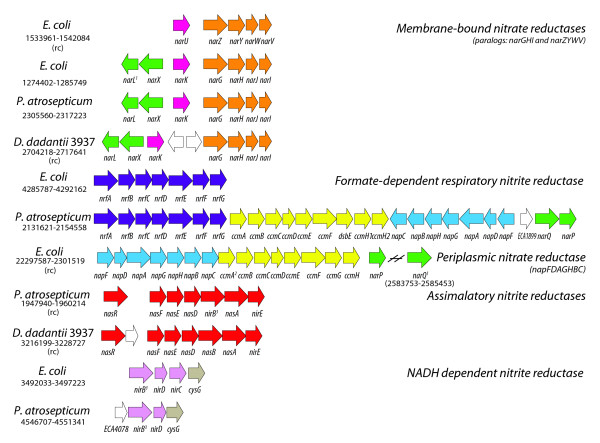
**Genomic architecture of genes involved with nitrate/nitrite metabolism in *E. coli*, *D. dadantii *and *P. atrosepticum***. Direction (forward or reverse complement indicated by (rc)) was selected to maximize collinearity. Colors are indicative of OrthoMCL grouping unless otherwise indicated by footnotes such that each color marks the genes associated with *E. coli *clusters and members of orthologous groups from *D. dadantii *3937 and *P. atrosepticum *SCRI1043 labeled with the same name. White genes are singletons with no orthologs in the other two organisms. 1. *narPQ *and *narXL *are paralogous components in *E. coli *and *P. atrosepticum *SCRI1043. The *narQ *locus is located elsewhere in the chromosome, 2. *ccmABCDEFGH*, type 1 cytochrome C biogenesis system contains a duplication of *ccmH *in *P. atrosepticum*, 3. OrthoMCL cluster that includes the *nirB *encoded large subunit from *E. coli*, a single protein from *D. dadantii *3937 annotated as *nirB*, and two paralogs from *P. atrosepticum *SCRI1043, annotated as *nirB *and *nasB*.

Comparative analysis reveals differences in genomic content of genes involved in nitrate/nitrite metabolism (Figure 4). The locus for the periplasmic nitrate reductase (*nap*), including part of the *ccm *operon and *narP*, is conserved between *E. coli *and *P. atrosepticum*, but is missing in *D. dadantii*. In *E. coli*, the genes for NarQ, and the respiratory nitrite reductase Nrf, which are also missing in *D. dadantii*, are not grouped in the same locus as found in *P. atrosepticum *(Figure 4). The *napABCDFGH *operon is not differentially expressed in *P. atrosepticum *or *E. coli *under the growth conditions used in our experiments. The *P. atrosepticum nrf *genes are > 3-fold up-regulated, while in *E. coli*, only *nrfB *shows a greater than 3-fold change. The *E. coli *NADH dependent nitrite reductase (*nirBDC-cysG *operon) is a cytoplasmic enzyme that does not produce a proton gradient, and is thought to be involved with detoxification of nitrite (Figure 4).

We believe *nirB *gene was erroneously assigned in *D. dadantii*, because this gene is part of a larger cluster conserved among the two phytopathogens that include a transcriptional regulator (*nasR*), an ABC transporter (*nasF*, *nasE *and *nasD*), two subunits of an assimilatory nitrate reductase (*nasB *and *nasA*), and an uroporphyrin-III C-methyltransferase (*nirE*). We hesitate to assign orthology of the *nas *systems between the phytopathogens because the genome context is not conserved beyond the *nas *subsystem itself, but it is clear that the phytopathogen loci are more structurally conserved (order and content) than either is with the *nir *system of *E. coli *(Figure 4).

*E. coli *has two characterized aerobic terminal reductases that are conserved in the phytopathogens. These are: the cytochrome bo3 type quinol oxidase (encoded by *cyoABCD*), which has a low affinity for O_2_, and the high-affinity bd-type cytochrome oxidase (encoded by *cydABCD*). The *cyoABCD *operon is down-regulated in *E. coli *and *P. atrosepticum*, but shows very little change in *D. dadantii*. The *cydABCD *operon is only up-regulated in *D. dadantii*. The cytochrome oxidase loci are regulated in part by ArcAB in *E. coli*, which may explain why *D. dadantii *shows the most divergent expression patterns. Additional Cyt bd type oxidases are encoded in *E. coli *(*appCB*) and *P. atrosepticum*, which may be strain-specific, as suggested by their genomic context and OrthoMCL clustering

*E. coli *encodes additional cytochromes with predicted roles in the electron transport chain. One of which, *yceJ*, is > 3-fold down-regulated in all three organisms under anaerobic conditions. The others show divergent patterns of gene expression. The gene *cybBD *(cytochrome b561) groups with *yodB in E. coli*, and single orthologs from both phytopathogens, of which only the *D. dadantii *ortholog is differentially expressed. The *E. coli cybC*, a pseudogene in strain MG1655, is a soluble cytochrome b562 of unknown function [45] but has orthologs that are divergently expressed in the phytopathogens (Additional File 8).

In *E. coli *there are 15 known dehydrogenases that donate electrons to the respiratory chain [46]. Some of these have already been mentioned, but most are conserved in the phytopathogen lineage (*sdhCDAB*, *mqo*, *glpD*, *ndh*, *nuo*). Only the operon encoding NADH dehydrogenase I, *nuo*, is differentially expressed in the phytopathogens while *ndh *remains unaffected by O_2_.

**Figure 5:**
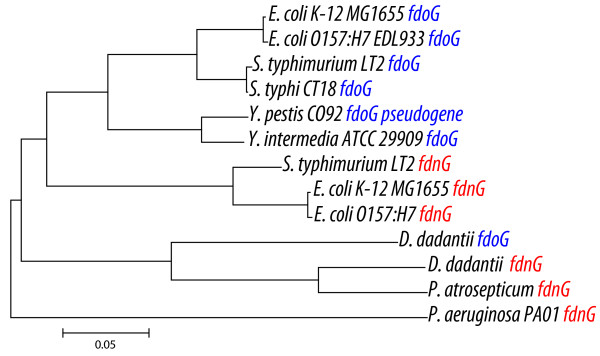
**Phylogenetic analysis of the major subunit of formate dehydrogenases from select enterobacteria**. Sequences of the major subunit of formate dehydrogenase from Fdh-O and Fdh-N were aligned using CLUSTALW. The tree was constructed using NJ with default parameters of MEGA 4.0.

The dehydrogenases involved in formate metabolism and reuse of dihydrogen have diverged in the phytopathogen lineage. These include the formate dehydrogenase N (Fdh-N, *fdnGHI*), which functions in the formate-nitrate respiratory chain and the structurally related formate dehydrogenase O (Fdh-O, *fdoGHI*). Fdh-O is expressed at relatively low levels independent of either O_2 _or nitrate availability and is thought to provide this critical activity during the transition to anaerobic growth [47]. *D. dadantii *encodes two formate dehydrogenases homologous to Fdh-O and Fdh-N, while *P. atrosepticum *has only one formate dehydrogenase. There is insufficient conservation of genome context to confidently assign orthology of these genes to either Fdn-N or Fdn-O. Phylogenetic analysis (Figure 5) of the major subunit genes of the two formate dehydrogenases showed that all three phytopathogen loci are more similar to each other than either of those found in *E. coli*, suggesting that none of them are orthologous to the *E. coli *loci. Furthermore, all the formate dehydrogenases in the phytopathogens are up-regulated even though nitrate is not present in the medium. In *E. coli*, expression of these genes is below detectable levels. An alignment of phytopathogen and *E. coli *sequences upstream of *fdnG *revealed a conserved promoter and an FNR binding site relative to the -42.5 position, however there are no NarL heptamer sites relative to the -77,-100,-109, or -124 positions in the phytopathogens.

There are two systems for the oxidation of hydrogen under anaerobic conditions in *E. coli*: the Tat dependent periplasmic uptake [48] hydrogenases hydrogenase 1 and hydrogenase 2 that are involved in hydrogen oxidation coupled to quinone reduction under anaerobic conditions. Like the FHL-type hydrogenases there are differences in the structure and content of these loci between *E. coli *and the phytopathogens (Figure 3). There are no phytopathogen orthologs for any genes of the *E. coli *hydrogenase 1 operon, *hyaABCDEF*. In *P. atrosepticum*, all hydrogenase related genes are clustered in a single chromosomal locus that OrthoMCL groups with *E. coli *hydrogenase 2. In *E. coli*, several genes of the hydrogenase 1 and 2 are up-regulated with others changing in a congruent though not statistically significant way. In *D. dadantii *and *P. atrosepticum*, all genes associated with the hydrogenase 2-like system are up-regulated.

It appears that much of the transcriptional variation between the phytopathogens and *E. coli *with respect to metabolic responses to O_2_, is mainly the result of changes in gene content, genome rearrangements of both important regulatory and metabolic components of the anaerobic stimulon, specifically in relation to the loss of the NarP regulon and a mutation in ArcB in *D. dadantii *and also of genes involved in nitrate/nitrite metabolism. Variation in the components of the respiratory formate dehydrogenases and hydrogenases in both phytopathogens, exhibit more complex evolutionary histories with equivalent functions carried out by paralogous or analogous systems, and many of these exhibit differential responses to O_2 _across these organisms. The only obvious energy metabolism subsystem present in the phytopathogens that is missing from *E. coli *involves butanediol fermentation.

Sequences of the *arcB *locus from *D. dadantii *strains in our lab confirm the *arcB *nonsense mutation (data not shown), but we have yet to confirm whether this mutation is found in *D. dadantii *3937 strains from other labs, although it is not present in any of the recently sequenced *Dickeya *species. Whether this mutation is laboratory derived or is a lineage specific event remains to be determined. Regardless, this strain has been successfully used to identify and test various pathogenicity related phenotypes *in planta *suggesting that it may not affect its ability to macerate host tissues.

### Stress responses

Analysis of the expression pattern of genes associated with various types of stress responses reveals interesting similarities and differences among the three organisms. Overall, the patterns are suggestive of a phytopathogen-specific oxidative stress response. For example, *ahpC*, *trxC*, *sodC *and *dps *encode bona fide virulence factors in bacterial pathogens [49-53] and they counteract damage due to reactive O_2 _species. Counterintuitively, these genes are up-regulated in the phytopathogens under anaerobic conditions and as expected, remain unaffected in *E. coli*. It is possible that prolonged growth in an O_2 _limited environment simulates a situation that soft-rotting bacteria experience prior to encountering the host oxidative burst. Our data suggest the possibility that both *P. atrosepticum *and *D. dadantii *may be able to anticipate and respond to a host induced oxidative environment before its onset, leading to the speculation that in these two phytopathogens the regulatory networks that govern responses to two opposite stressors (anaerobic stress and oxidative stress) may be linked to increase chances of survival in the plant environment. Such anticipatory responses are proposed to occur in organisms living in environments that change in predictable ways [54]. The phytopathogen-lineage specific up-regulation of both the *narGHJI *genes and the nitrate-dependent formate dehydrogenase genes (*fdnGHI*) involved in respiratory nitrate reduction, despite the absence of nitrate in our growth media, may also be regarded as an anticipatory response in view of the fact that nitrate is an abundant anion in plant. Similar to *E. coli*, it is possible that a complex regulatory network that includes PecS [55], OxyR [56], FNR, (represses *sodC in E. coli*), RpoS (induces *sodC*) as well as regulatory elements of nitrate metabolism may be involved.

Some oxidative stress responsive genes that are down-regulated in the phytopathogens, as expected in an anaerobic environment, are either lineage-specific (*ohrR and ohr*) or strain-specific (*indABC, vfmABCDE*) and are not present in *E. coli*. The expression pattern of several other genes associated with oxidative stress is similar in *E. coli *and *P. atrosepticum*, but differs in *D. dadantii *and for two of these genes, *sodA *and *tpx*, ArcA-mediated regulation has been demonstrated for the *E. coli *ortholog [57]. Orthologs of universal stress proteins and cold shock proteins, which are involved in the response to a variety of environmental stresses in enterobacteria [58-62], are up-regulated in one or both phytopathogens but remain unaffected in *E. coli *(see Figure 2)

### Metal transport systems

A variety of transition metals including iron, manganese, nickel, zinc and copper are required by bacteria for the activity and stabilty of proteins, including FNR and most of the respiratory enzymes. A large number of genes are devoted to their acquisition, uptake, storage and efflux in order to maintain homeostasis. Since several of the anaerobic respiratory enzymes require a different complement of metals (e.g. nickel) than the aerobic respiratory enzymes, and because unbound iron oxidation states is readily influenced by the O_2 _status of the growth media, we are not surprised to observe transcriptional changes for associated genes, and some of them are described below.

Each of the three organisms up-regulates at least one nickel uptake system (*nikABCDE *in *E. coli*, *hoxN *in the phytopathogens) indicating an increased requirement for this metal during anaerobiosis. In *E. coli *the NikABCDE system transports nickel for the NiFe hydrogenases. Genes encoding transport systems for copper and zinc largely show phytopathogen-specific responses. For example, the Cus metal efflux system (*cusCFBA *operon) is up-regulated in both phytopathogens and is down-regulated in *E. coli *whereas, the *copA *gene is down-regulated in both phytopathogens, but not in *E. coli*. The Cus system and CopA are associated with copper homeostasis under anaerobic and aerobic conditions respectively, in *E. coli *[63]. Similarly, zinc uptake systems (*znuABC*) are down-regulated only in the phytopathogens. Additionally, both phytopathogens down-regulate transcripts (> 3-fold) for an ABC transport system predicted to transport zinc (according to the *D. dadantii *annotations). Genes for this system are not present in *E. coli*. All three organisms encode a zinc uptake regulator (Zur), the gene for which is down-regulated only in *D. dadantii*.

All three organisms have a substantial number of genes involved in synthesis and transport of iron chelating siderophores and other iron-containing compounds. Overall, these subsystems are down-regulated in the phytopathogens, and to a lesser extent in *E. coli *suggesting that the phytopathogens may have a reduced demand for iron during anaerobiosis or may reduce iron levels to avoid damage in the anticipated oxidative environment of the host. Many of these genes are likely regulated by Fur in all three organisms.

Genes that belong to the same ortholog group do not necessarily synthesize the same siderophore although they might be able to transport some of them. For example, OrthoMCL clusters genes for synthesis of the *D. dadantii *siderophore, chrysobactin, with enterobactin synthesis genes of *E. coli*, but the siderophores are distinct [64]. *D. dadantii *does not synthesize enterobactin, although it is capable of uptake and utilization of exogenous enterobactin [65]. The OrthoMCL clusters also include single members from *P. atrosepticum*, but none of the genes share extended conserved genomic context across organisms, and there is no data on whether *P. atrosepticum *produces enterobactin, chrysobactin or another siderophore using these genes. It is also established that some bacteria can "steal" siderophores from their neighbors as seen in *P. atrosepticum *which is unlikely to produce achromobactin, but may be able to uptake and transport it via genes orthologous to the *D. dadantii cbrABCD *[66]. A detailed comparison of the *D. dadantii *and *P. atrosepticum *iron homeostasis systems is found in Franza and Expert [64].

Genes for several iron transporting ABC transport systems shared only between the phytopathogens are all largely down-regulated in both organisms (for example OrthoMCL groups 3161-3163, *yfeABCD*, *sfuABC *[67]). In *E. coli*, under anaerobic conditions uptake of ferrous iron is expected to increase relative to oxidized ferric iron. In line with this expectation, the *feoAB *genes that are involved in transport of ferrous iron are up-regulated in *D. dadantii*. However, in the Kang et al. experiments, neither the *efeBOU *genes nor the *feoAB *genes, which encode ferrous iron transporters were up-regulated. Several genes encoding iron storage proteins are up-regulated in the phytopathogens and remain unaffected or are down-regulated in *E. coli *(see Additional File 7). These include the *bfr *gene, encoding a bacterioferritin which contributes differentially to the virulence of *D. dadantii *depending on the host [68] and *dps *which encodes a ferritin-like protein, Dps, with pleiotropic functions. *D. dadantii *also up-regulates transcripts for a second strain-specific Dps-like protein (ABF-0015905).

### Other transporters

Most of the transporters for amino acids are down-regulated in the phytopathogens, and unaffected in *E. coli*. Only one gene (sulfate transporter; *yfbS*) shows > 3-fold congruent up-regulation between the phytopathogens and a sodium-serine transporter gene (*sst*) is up-regulated both in *E. coli *and *P. atrosepticum*. Even though OusA has been implicated in anaerobiosis in *D. dadantii *[69], we did not detect changes in expression for its gene in our experiments.

### Cell-wall degradation

In *P. atrosepticum *and *D. dadantii*, pathogenicity is largely due to their capacity to depolymerize plant cell wall polymers including cellulose, hemicellulose and pectic substances, as well as other components such as lignin and proteins, through the coordinate production of multiple cell wall degrading enzymes (CWDE). Because of their indispensable role in pathogenicity, expression of CWDE is strictly regulated at the transcriptional level by multiple regulators and further fine tuned by environmental factors including pH, osmolarity and O_2 _concentrations all of which influence the successful onset of disease symptoms [70]. Anaerobic regulation in the presence of an inducer has been demonstrated for *pelA*, *pelD*, *pelE *and *pelL *[71] in *D. dadantii*. Except for *pelD *none of these four genes has an ortholog in *P. atrosepticum *(see Additional File 7). As expected for cells grown in non-inducing conditions, all the reported pectate lyase genes (*pelA *to *E*, *L*, *Z*, *I*) are repressed in *D. dadantii*. This trend is not seen in *P. atrosepticum *where in fact, one gene, *pelB*, is up-regulated. The only CWDE-encoding gene that is down-regulated in *P. atrosepticum *is a pectin lyase, *pnl*, which is a member of a complex ortholog group. It is interesting to note that a *D. dadantii *-specific gene, *xynA *(ABF-0019026), encoding a putative endoxylanase, characterized in a related corn pathogen [72], is > 3-fold up-regulated.

### Phytopathogen secretion systems

Both *D. dadantii *and *P. atrosepticum *encode a diverse collection of secretion systems, several of which are known to play key roles in interaction with plant hosts. The T6SS shows one of the most dramatic divergent expression patterns observed in our experiments. The T6SS mediates secretion of proteins encoded within repetitive clusters of genes found distributed throughout the genome, often, though not reliably, annotated as *hcp *and *vgrG*. OrthoMCL clusters paralogs of each type into two groups. The *hcp *cluster includes three members from *P. atrosepticum *and two from *D. dadantii*. The *vgrG *cluster includes three members from *D. dadantii *and five from *P. atrosepticum*. The *D. dadantii *T6SS is down-regulated in the absence of O_2 _and the *P. atrosepticum *T6SS is up-regulated. A previous report demonstrated that the *P. atrosepticum *T6SS cluster and proteins secreted via the T6SS (four *hcp *genes and three *vgrG *genes) are induced by plant host extracts [73,74]. Mutants of two genes, believed to correspond to a structural component of the secretion apparatus (*vasK*) and a sigma-54 dependent regulator (*vasH*), showed increased virulence relative to wild-type, a phenotype attributed to increased growth (higher density) and associated increases in pectic enzyme production in the mutants. In our analyses, *vasK *and *vasH *are up-regulated, and the corresponding orthologs in *D. dadantii *are both down-regulated, typical of the T6SS clusters as a whole. All members of both *hcp *and *vgrG *groups are up-regulated and exhibit an expression pattern congruent with the T6SS in each organism.

Under the anaerobic conditions used in our experiments, genes for type I secreted proteases (PrtA, PrtB, PrtC, PrtG) and for their accessory proteins (PrtD, PrtE and PrtF) show a similar trend as the CWDE; they are down regulated in *D. dadantii*, and remain unaffected in *P. atrosepticum*. The Type II secretion system (T2SS) is responsible for secretion of CWDE as well as several other targets [75]. In addition, it has been linked to iron homeostasis in *D. dadantii *with interactions between inner membrane components of the T2SS and the machinery for achromobactin synthesis [76]. *E. coli *has an orthologous secretion system that is not expressed in wild-type *E. coli *strains but is functional in *hns *mutant strains [77], and the corresponding genes are unaffected in *E. coli*. Genes associated with the T2SS are nearly all down-regulated in *D. dadantii *and unaffected in *P. atrosepticum*, where absolute expression levels remain high regardless of O_2 _availability. *D. dadantii *also encodes a second locus similar to genes of a T2SS, which is associated with targeting proteins to the outer membrane [78]. Several genes from this system in *D. dadantii *are also down-regulated. Both phytopathogens encode a Type III secretion system (T3SS), a syringe-like apparatus employed by numerous Gram-negative pathogens to inject bacterial proteins into host cells. Although the T3SS is required for full virulence in *D. dadantii *[79] and *P. atrosepticum *[80], far fewer secreted effector proteins have been identified in soft-rot associated pathogens than many other bacteria, and some pathogenic *Pectobacterium *lack a T3SS altogether [81]. The *D. dadantii *T3SS has also been implicated in multicellular behavior and biofilm formation [82]. Genes associated with the T3SS are largely unaffected in both *D. dadantii *and *P. atrosepticum*, with low absolute expression levels with and without O_2_. However, in *D. dadantii *several related genes are down-regulated including, *hrpS*, which encodes a σ54-enhancer binding regulatory protein, two secreted harpin genes, *hrpN *and *hrpW *and *dspE *encoding a T3 secreted effector. None of these genes show a similar response in *P. atrosepticum*. Genes that encode a putative two partner secreted adhesin and its associated activator/transporter constitute a complex OrthoMCL group that has two *D. dadantii *genes and three *P. atrosepticum *genes (includes *hecA/B*). Only the *P. atrosepticum *orthologs are up-regulated under anaerobiosis. In *E. chrysanthemi *strain EC16 a role for HecA in early pathogenesis has been suggested [83].

### Chemotaxis

Methyl-accepting chemotaxis proteins (MCPs) transduce environmental and cellular signals to the flagella [84]. The *D. dadantii *genome has 45 genes that encode proteins whose products are annotated as MCPs, while there are 36 such genes in *P. atrosepticum*. The C-terminal signal transduction domain of MCPs is highly conserved across all members of the family, while the N-terminal sensory domain varies extensively. This complicates reliable prediction of orthology. In our OrthoMCL analysis, 12 out of 15 *D. dadantii*-specific MCPs and one of 5 *P. atrosepticum*-specific MCPs are differentially expressed. Of the MCPs shared between the phytopathogens 17 are differentially expressed in *D. dadantii *and 9 in *P. atrosepticum*. Setting aside the potential errors with prediction of orthology, there is clearly an O_2_-availability regulated motility response in both *D. dadantii *and *P. atrosepticum*.

Though not an MCP, the *E. coli *Aer has been associated with aerotaxis via sensing of cellular redox potential using an FAD cofactor [85]. The phytopathogens each have three homologs that show similarity to Aer throughout the entire length of the alignment (*aer1*, *aer2 *and ABL-0063893 in *P. atrosepticum *and *aer1*, ABF-0014726 and ABF-0014843 in *D. dadantii*). Both phytopathogens include at least one putative aerotaxis receptor up-regulated and one down-regulated under anaerobic conditions. Transcripts of the *E. coli aer *gene decrease, though not statistically significantly, during anaerobiosis.

### Flagella, motility and attachment

In the soft-rot pathogens, the contribution of flagella to motility is important for virulence [86-88]. Genes encoding regulatory elements of flagellar assembly as well as some of the genes of the flagellar apparatus are affected under anaerobic conditions in one or both phytopathogens. None of the differentially expressed genes show a similar trend in both phytopathogens (see Additional File 7) suggesting that these genes may be regulated differently during anaerobiosis in these two phytopathogens.

### Polysaccharides

Genes encoding enzymes that produce a range of polysaccharides such as lipopolysaccharides (*rfa *and *waa *genes), exopolysaccharides/O-antigens (*wza *and *rfb *genes), enterobacterial common antigen (*wec *and *rff *genes) and membrane derived oligosaccharides such as periplasmic glucans (*opg *genes) play important roles in virulence, adhesion, resistance to host-derived compounds and are considered virulence factors in *P. atrosepticum *and/or *D. dadantii *[89-93]. In our experiments, the expression of these genes is mostly unaffected in *P. atrosepticum *and some of them are down-regulated in *D. dadantii*.

### Toxins

The *P. atrosepticum *genome contains a cluster of 9 *cfa *and *cfl *genes that are > 3- fold up-regulated in the absence of O_2_. They encode a putative polyketide biosynthesis system predicted to synthesize a compound similar to coronafacic acid, a component of the coronatine phytotoxin produced by *P*. syringae [94], and mutations in the *P. atrosepticum *genes dramatically reduces virulence on potato. A similar gene cluster was recently characterized from a phytopathogenic *Streptomyces*, with mutants in the polyketide synthesis system showing reduced virulence on tobacco [95].

*D. dadantii *is pathogenic to pea aphids under laboratory conditions and this trait appears relatively widely distributed among species of enterobacteria [96]. Deleting a cluster of four genes encoding proteins similar to cytolytic delta-endotoxins from the gram-positive entomopathogen *Bacillus thuringiensis *significantly reduced virulence of *D. dadantii *on aphids. These four genes (typically expressed as a single transcriptional unit) are up-regulated in our experiments. Interestingly these genes were regulated by many of the same regulators that control expression of virulence factors for the plant host but in opposite directions [97].

### Pathogenicity-associated transcriptional regulators (TR)

In *P. atrosepticum *and *D. dadantii*, several regulators coordinate expression of virulence factors in response to environmental or physiological conditions [98-103]. O_2 _dependent modulation has been demonstrated for very few virulence factors or associated regulators.

In our experiments, *D. dadantii *and *P. atrosepticum *each appear to have at least one O_2_-responsive strain-specific global regulatory gene whose expression is influenced by anaerobiosis. They are a gene for PecM [103-107] in *D. dadantii *and that for RdgB in *P. atrosepticum *[108,109]. Beside these, the PecS repressed *expI *gene [110] coding for the LuxI homolog in *D. dadantii *for the production of AHL, the *expR *gene that activates PecS and which encodes the AHL receptor are down-regulated only in *D. dadantii*, and their orthologs are unaffected in *P. atrosepticum*. Interestingly, many regulators shared between the phytopathogens are differentially expressed in a lineage-specific pattern that may indicate regulatory divergence for these loci. It is possible that at least some of these regulators respond to host-derived signals under the O_2_-limiting and inducing conditions encountered within the plant.

Most of the other global regulators of virulence including KdgR [111,112], Crp [99,101] and Fur (*fur *shows statistically significant up-regulation but only minimal (~1.7) fold-change), that are highly conserved between the phytopathogens are unaffected in either organism. They all show moderate levels of expression regardless of O_2 _availability. Although transcription of several KdgR target genes is affected in our experiments, the involvement of KdgR is unlikely to account for the change in expression for these targets since the inducer 2-keto-3-deoxygluconate (KDG), a pectin degradation compound was not present in our medium. Furthermore other than CWDE genes mentioned above, which are members of complex overlapping regulons, none of the co-regulated transporter genes such as KdgMN and *togMNAB *are affected in our experiments.

### Non-protein coding genes in the anaerobic stimulon

#### Small RNAs

Small RNA genes are increasingly recognized as important global regulators of diverse biological processes, but relatively few small RNAs have been directly linked to the response to O_2_, even in *E. coli *where small RNAs have been most extensively investigated. In *D. dadantii*, a total of 12 small RNA genes were differentially expressed with a 3-fold or greater magnitude, and in *P. atrosepticum*, 6 small RNA genes met these criteria (Table 3). Small RNA genes that show a consistent anaerobic response in both phytopathogens include *fnrS*, a gene known to be O_2_-responsive in *E. coli*, as well as *ffs *(4.5S) and *ssrS *(6S), both of which are conserved in *E. coli*, though previously unlinked to the anaerobic stimulon and unaffected in the Kang et al. experiments. FnrS and ArcZ have been implicated in anaerobic regulation of a variety of targets in *E. coli*. In *E. coli *transcriptional activation of *fnrS *by FNR during anaerobiosis leads to translational repression of *cydDC*, *metE*, *sodA*, *sodB*. FnrS also activates at least one target gene (*yhaO*); [113,114]. In the phytopathogens, *fnrS *is > 3- fold up-regulated in both *D. dadantii *and in *P. atrosepticum*. In *E. coli*, a second small RNA, ArcZ, is important under anaerobic conditions and is regulated by ArcAB [115]. In our experiments, *arcZ *is detected as differentially expressed in *D. dadantii*, but not in *P. atrosepticum*.

**Table 3:** List of small RNAs that are O_2_-responsive in at least one of the two phytopathogens *D. dadantii *and *P. atrosepticum*

ASAP Feature ID			Gene Name	Fold Change			References
*D. dadantii*	*P. atrosepticum*	*E. coli*		*D. dadantii*	*P. atrosepticum*	*E. coli*	
ABF-0061315	ABL-0064934		*arcZ*	1.9	-1.2*		[115]
ABF-0061324	ABL-0061410	ABE-0001579	*ffs*	12.8	4.3	1.4*	[116-118]
ABF-0174125	ABL-0064933		*fnrS*	28.3	52.4		[113,114]
ABF-0061309	ABL-0064917		*glmY*	3	1.4*		[119-123]
ABF-0061313	ABL-0064935		*glmZ*	-0.8*	1.9*		[119-123]
ABF-0061316	ABL-0060542	ABE-0010269	*rnpB*	2.7	1.2*	1.6*	[12]
ABF-0061322	ABL-0061263		*rsmB*	3.7	1.2*		[124-126]
ABF-0061325	ABL-0062736		*ryeA*	2.3	-2.1		[127,128]
ABF-0061326	ABL-0062735		*ryeB*	5.4	-2.3		[127,128]
ABF-0061311	ABL-0063956		*rygA*	6.3	-2.0*		[129,130]
ABF-0061314	ABL-0060225	ABE-0012621	*spf*	16.3	-1.1*	2.08*	[131]
ABF-0061318	ABL-0060877		*sraF*	0.6*	-3		[132]
ABF-0061317	ABL-0060677	ABE-0009556	*ssrS*	5.2	2	1.75*	[133]
ABF-0061323	ABL-0061273		*tff*	-1.8	-1.1*		[134]

This list of small RNA genes are differentially expressed in one or both phytopathogens. Where there is a gene in *E. coli *in the group, the fold change is included. Statistically insignificant values are marked with an asterisk.

Several other small RNA genes show fairly compelling evidence of differential transcriptional regulation between the two phytopathogens including *spf *(Spot 42), *rygA/omrA *and *ryeA*, and possibly *glmY*, *glmZ*, and *rsmB*. In *D. dadantii*, one up-regulated small RNA corresponds to the *spf *gene, an ortholog of the *E. coli *Spot 42 small RNA, which plays a role in anti-sense mediated down-regulation of the third gene (*galK*) of the galactose operon [116,135], and whose expression is known to be affected by carbon source available in the media and is cAMP-CRP responsive [136]. The corresponding gene in *P. atrosepticum *does not change expression, rather levels are intermediate in both conditions, similar to the *E. coli *ortholog in the Kang experiments. The *rygA *gene is > 3-fold up-regulated in *D. dadantii*. The *P. atrosepticum *ortholog is not differentially expressed, and trends in the opposite direction. Targets of the two *E. coli *orthologs, neither of which have previously been implicated in anaerobiosis, include both transcriptionally up and down regulated genes many of which are involved with cell surface structures or functions. They also negatively regulate *fepA *and *fecA*, two genes associated with iron homeostasis, and fimbrial genes associated with adhesion and biofilm formation [130]. The *ryeB *RNA is up-regulated in *D. dadantii *and down-regulated in *P. atrosepticum*. In *E. coli*, RyeB interacts with RyeA, encoded on the opposite strand, to mediate RNAse III-dependent cleavage [127] and is known to be pH-responsive in O_2_-limited conditions [128]. We detect *ryeA *as differentially expressed in *D. dadantii*, although not in *P. atrosepticum*, where it trends in the same direction as *ryeB*. GlmY, a small RNA implicated in amino-sugar metabolism, is detected as up-regulated in *D. dadantii *and unaffected in *P. atrosepticum*. Amino sugars are important precursors of the peptidoglycan and lipopolysaccharide components of the cell wall. The *rsmB *gene is highly expressed under both aerobic and anaerobic conditions in *D. dadantii *and *P. atrosepticum*. It is up-regulated in the absence of O_2 _only in *D. dadantii*. This gene, like several others, was also not present on the Kang et al. arrays. RsmB has been linked to production of extracellular enzymes, quorum sensing and T3SS in *Dickeya *as well as in a related species [124-126]. This collection of lineage-specific expression patterns suggests that altering regulation of small RNAs may be a particularly labile mechanism of regulatory diversification.

## Conclusions

We investigated the transcriptional response to O_2 _under simple controlled laboratory conditions for two soft rot-associated phytopathogenic enterobacteria to begin to enumerate the regulatory and metabolic networks associated with a key environmental parameter that impacts the interaction of these organisms with plant hosts, and to explore the extent of regulatory divergence that occurs among enterobacteria. We analyzed data from *D. dadantii *and *P. atrosepticum *individually, and compared them to each other, as well as the model organism *E. coli *K12, using predicted gene-by-gene orthology and by grouping related genes into subsystems. The latter approach provides insights into larger scale patterns of conserved and lineage-specific biological processes regulated by O_2 _availability.

The O_2_-responsive stimulon for each organism is large, and includes genes conserved across the family enterobacteria, as well as lineage-specific and organism-specific genes that were likely acquired through lateral gene transfer events. Some conserved genes show a conserved response to O_2_, but others vary across organisms in the magnitude or even direction of response. *D. dadantii *and *P. atrosepticum *are more closely related to each other than to *E. coli *K12, and overall, their gene expression profiles are more conserved in terms of total number of orthologous genes (including those not shared with *E. coli*) responding in a congruent way, and in the proportion of genes shared across all three organisms responding in a congruent way.

A subset of genes show a different trend, with the expression profile more similar between *E. coli *and *P. atrosepticum*, with *D. dadantii *behaving differently. We attribute many of these to the likely deficiency of ArcAB regulation in *D. dadantii *where the sensor kinase of this two-component regulatory system is a pseudogene; however, it is not possible from these experiments to rule out that ArcA may be partnered with a different signal transducer, opening up the possibility that existing binding sites for ArcA could be coopted to respond to an entirely different signal.

A relatively small number of conserved genes are > 3-fold up-regulated or down-regulated in all three organisms. While these include some known components of the well characterized *E. coli *anaerobic stimulon, other important components are missing from this conserved core. Detailed investigation of the orthology relationships and the subsystem-based approach reveal a broader group of processes implicated in a net conserved response to O_2_, but with variation across the organisms in the number of functionally redundant paralogs and/or non-paralogous isofunctional subsystems, which we refer to as changes in subsystem architecture. For example, hydrogenases are highly up-regulated under anaerobic conditions in all three organisms, but the genes involved show complex homology relationships, involving duplications, and or deletions, as well as genome rearrangements, that obscure the common response. We detailed these types of relationships and the associated expression patterns for subsystems involved with regulation, metabolism and a variety of processes associated with interactions of the phytopathogens with plant hosts.

Our results indicate that the O_2_-responsive gene network includes a variety of virulence and pathogenicity-relevant processes including secretion, response to environmental stress, metal homeostasis, and taxis. Further experiments aimed at investigating the specific role of O_2 _regulation of these biological processes may be fruitful. Several virulence-associated subsystems exhibit strikingly divergent behavior in the two phytopathogens, most notably the T6SS. More subtle differences, like down-regulation of the complete complement of pectate lyases in *D. dadantii*, which are largely unaffected in *P. atrosepticum*, may lead to insights into the differences in virulence of these two phytopathogens under O_2_-limited conditions, but this requires exploration under a broader number of experimental conditions. The experimental conditions explored here are very limited, and the number of genes in the plant-pathogen anaerobic stimulons will only increase as additional variables (carbon sources, nitrate availability, intermediate O_2 _levels, time series of shifting O_2 _availability, etc.) are investigated.

The genes differentially expressed in one or both phytopathogens include known targets of regulators associated with quorum sensing, oxidative stress, iron homeostasis, nitrate/nitrite, and carbohydrate availability, as well as the established key regulators of anaerobiosis under the experimental conditions we chose, FNR and ArcAB. The underlying regulatory network behind the O_2_-responsive stimulon we have described is complex, involving a larger number of regulators, and it clearly differs between the two phytopathogens. Further dissection of this network bioinformatically will certainly require simultaneous consideration of a large number of regulators. We do not attempt a comprehensive dissection in this paper. We observe that at least one known key regulator of virulence genes, PecM, is among the genes differentially affected between *D. dadantii *and *P. atrosepticum*.

Several small regulatory RNAs are also differentially expressed under aerobic and anaerobic conditions, including ones that are found in *E. coli*, but have not previously been characterized as O_2_-responsive. These expression patterns should be experimentally validated using additional techniques, both because of their novelty, and because small genes may be particularly subject to measurement errors using arrays.

Many of the organism-specific and phytopathogen-specific genes in the anaerobic stimulon were likely acquired through lateral gene transfer events. These genes may have become a part of the stimulon in a variety of ways. A lateral transfer can include regulatory elements from the donor that also function in the recipient or a laterally acquired gene could be integrated into the recipient genome in a way that makes use of native O_2_-responsive regulatory elements. Alternately, evolutionary events (point mutations, rearrangements, further lateral transfers in the same region) subsequent to acquisition of a gene could render it O_2_-responsive. We expect there to be examples of all of these, but further examination awaits comparison with additional representatives of each genus from ongoing genome projects that will permit more precise definition of the boundaries of laterally acquired elements and reconstruction of the ancestral regulatory states. Finally, these experiments addressed only a single representative of each species, and further investigation will be required to determine whether the expression patterns we observed here are typical of each species, and which aspects of the anaerobic stimulon vary within each species.

## Methods

### Bacterial growth and RNA extraction

We grew three replicates each of *Dickeya dadantii *3937 and *Pectobacterium atrosepticum *SCRI1043 in MOPS minimal medium (purchased from Teknova, Inc.) supplemented with 0.1% glucose at 30°C and 23°C respectively. Overnight cultures of each bacterium were diluted to an O.D._600 _of 0.05 in fresh medium and cultured in a gas sparging system [137] apparatus described in Kang et al.) that permits precise control over the mixtures of O_2_, N_2 _and CO_2_. Cultures were grown to early log phase under aerobic (70% N_2_, 25% O_2 _and 5% CO_2_) and anaerobic (95% N_2 _and 5% CO_2_) conditions. 20 ml samples were collected in tubes containing 2 ml phenol-ethanol. RNA was extracted using the hot-phenol method [138]. Quality of RNA samples was assessed using the Agilent Bioanalyzer 2100 nanochip system (Agilent Technologies).

### Microarray design and hybridization

334647 genome-specific probes for the *D. dadantii *and 344859 probes for the *P. atrosepticum *genomes were selected using chipD (target melting temperature 78°C, target probe length 40 to 70 -mers, interval size 12) [139] and oligonucleotide arrays were synthesized by Nimblegen Inc. Procedures described in the Nimblegen Arrays User's guide (http://www.nimblegen.com/products/lit/05434505001_NG_Expression_UGuide_v6p0.pdf) were followed for cDNA synthesis, labeling and hybridization. Arrays were scanned at 532 nm and signals were extracted using NimbleScan software (NimbleGen, Inc.).

### Analysis of gene expression data

Signals from the 3 replicates of the hybridization experiments from each organism were normalized using RMA [129] implemented in the NimbleScan software and imported into a custom MicroSoft Access database. Normalized signal intensities for each set of three replicates are in good agreement (R^2 ^ranging from 0.92-0.99). The median of signals from multiple probes (from coding and non-coding strand) for each individual gene was calculated using R, from which log_2 _expression values were derived. To determine directional changes in gene expression, log_2 _ratios were determined by calculating the difference between the log_2 _median anaerobic signal and log_2 _median aerobic signal.

To identify differentially expressed genes between aerobic and anaerobic growth, an empirical Bayesian analysis, EBArrays [18] was executed within the free statistical analysis software package R [140] and Bioconductor v2.1 [141]. The posterior probability for each pattern was calculated using a hierarchical log-normal normal expression model with the conditional false discovery rate (cFDR) at 0.01 to determine the appropriate threshold (cFD(τ)). The *E. coli *WT data set for aerobic and anaerobic conditions derived from Kang et al. was reanalyzed to identify differentially expressed genes as described above. The critical thresholds for the three data sets are as follows:

*D. dadantii *: 0. 8734342, *P. atrosepticum*: 0.9067187, *E. coli*: 0.9289846. Throughout the manuscript, we have used the term "differentially expressed" to denote genes that qualify our permissive criterion namely the probability for observed differential expression is higher than the critical threshold, for the dataset, when the conditional false discovery rate is set at 0.01 and the prefix "highly" is used to denote differentially expressed genes whose transcripts show more than a 3-fold change between the conditions (stringent criteria). Genes whose transcript abundance is higher under anaerobic conditions are referrred to as "up-regulated" and those with lower transcript abundance under anaerobic conditions are referred to as "down-regulated". In some instances, fold change values for poly-cistronic operons that are conserved across all three organisms are averaged across genes to simplify our analyses. Additional File 9, Additional File 10, and Additional File 11 contain the complete datasets for *D. dadantii*, *P. atrosepticum *and *E. coli*, respectively.

### Analysis of expression data for non protein coding RNA (small RNA)

Our oligonucleotide arrays included probes tiled across the entire genomes for both *D. dadantii *and *P. atrosepticum*. Thus, we are also able to analyze the O_2_-response for genes typically not included in gene expression arrays, like those that encode small RNAs (sRNAs), even if they were not present in the original genome annotations. OrthoMCL considers only protein-coding genes, and there are not comprehensive predictions of orthology for non-coding RNAs (ncRNAs) or tRNAs in the ASAP database (the sRNAbase [142] has predictions for 34 small RNAs in *P. atrosepticum*) so we do not include them in the cross-species analyses above. Instead, we used BLASTn to find orthologs in both phytopathogens on a case-by-case basis. Most small RNA genes discussed in the results and discussion are missing or incorrectly annotated in at least one of the two phytopathogen GenBank genome sequences. We have corrected them in the ASAP database. These RNAs are short, and consequently, inferences about expression patterns are based on a relatively small number (typically around 10) of probes. We manually investigated probe behavior consistency in most cases, and found the expression patterns persuasive. All small RNA genes detected as differentially expressed in either organism are shown in Table 3.

### Comparative analysis

Sequences and annotations for predicted protein-coding genes for *D. dadantii *3937, *P. atrosepticum *SCRI1043 and *E. coli *were obtained from the ASAP database [143] and clustered using OrthoMCL [19] using default parameters. OrthoMCL clustered genes from each of the three organisms into simple or complex ortholog groups depending on whether one or more than one ortholog is present for the gene in the organsims being compared as described using the following example. *E. coli *encodes paralogous genes for fumarase, *fumA *and *fumB *that are homologous to the gene identified as *fumA *in the phytopathogens. All of these four genes are clustered together in a single orthologous group (complex group) by OrthoMCL. The third *E. coli *fumarase isozyme, encoded by *fumC *has a simple 1-1-1 relationship with *fumC *of *D. dadantii *and *P. atrosepticum *and these three genes are clustered together as a different group (simple group). Strain-specific genes do not belong to any OrthoMCL group. Additional File 8 lists all protein-coding genes from all three organisms, OrthoMCL group identifiers, and counts of the number of members of the group from each organism along with a short form of the experimental data results. Operon structures and known and predicted regulator binding sites for *E. coli *were obtained from EcoCyc [144] unless otherwise noted.

## Competing interests

The authors declare that they have no competing interests.

## Authors' contributions

All authors contributed to the overall concept and in the writing of the manuscript. LB and VB performed the experiments designed by JA, LB, JDG and NP. LB and JA participated in statistical analysis. PL performed the OrthoMCL analysis. All authors read, contributed to revisions of and approved the final manuscript.

## Supplementary Material

Additional file 1 **List of orthologous genes in *D. dadantii *and *P. atrosepticum *that are detected as differentially expressed using stringent criteria (cFDR = 0.01 and fold change > 3)**. Click here for file

Additional file 2 **List of simple orthologs in the phytopathogens (*P. atrosepticum and D. dadantii*) that are detected as differentially expressed (cFDR = 0.01) in both phytopathogens, but one or the other, or even both have fold changes < 3**. Orthologs that show congruent expression patterns as well as those that show divergent expression patterns are listed. Click here for file

Additional file 3 **List of simple orthologs in all three organisms (*E. coli, P. atrosepticum and D. dadantii*) that are detected as differentially expressed in at least one using stringent criteria (cFDR = 0.01 and fold change > 3)**. Click here for file

Additional file 4 **List of genes shared with *E. coli *and only one of the phytopathogens that are detected as differentially expressed using stringent criteria (cFDR = 0.01 and fold change > 3)**. Click here for file

Additional file 5 **List of strain-specific genes in *D. dadantii *that are detected as differentially expressed using stringent criteria (cFDR = 0.01 and fold change > 3)**. Click here for file

Additional file 6 **List of strain-specific genes in *P. atrosepticum *that are detected as differentially expressed using stringent criteria (cFDR = 0.01 and fold change > 3)**. Click here for file

Additional file 7 **Fold change patterns for *P. atrosepticum *and *D. dadantii *genes implicated in virulence and for their orthologs in *E. coli *predicted by OrthoMCL**. Anaerobic growth in the presence of glucose alters expression of virulence-associated genes in both *P. atrosepticum *and *D. dadantii*. This includes genes associated with cell wall degradation and the Out system (down-regulated in *D. dadantii *only), T6SS (down-regulated in *D. dadantii *and up-regulated in *P. atrosepticum*), iron uptake (down-regulated in both), methyl-accepting chemotaxis and regulators associated with virulence. Fold change patterns and genome order are as follows: *E. coli, P. atrosepticum and D. dadantii *(see key within the figure). Each orthologous group of genes is represented by three blocks colored by fold change (dark blue: down-regulated, fold change > 3; light blue: down-regulated, fold change < 3; bright yellow: up-regulated, fold change > 3; dirty yellow: up-regulated, fold change < 3; black: no change in expression, X: ortholog absent in that organism). Genes are represented by their symbols, or by ASAP feature IDs (especially for strain-unique genes) or by OrthoMCL group designations ("G" followed by number). Click here for file

Additional file 8 Complete OrthoMCL analysis. Click here for file

Additional file 9 **Normalized signals, fold changes and significance for features in *D. dadantii *3937**. Click here for file

Additional file 10 **Normalized signals, fold changes and significance for features in *P. atrosepticum *SCRI1043**. Click here for file

Additional file 11 **Normalized signals, fold changes and significance for features in *E. coli *K12-MG1655**. Click here for file
