# Supplementary figures and images for: Evolution of the metabolic and regulatory networks associated with oxygen availability in two phytopathogenic enterobacteria
Source: BMC Genomics. 2012 Mar 22;13:110. doi: 10.1186/1471-2164-13-110 (PMC3349551; doi:10.1186/1471-2164-13-110)

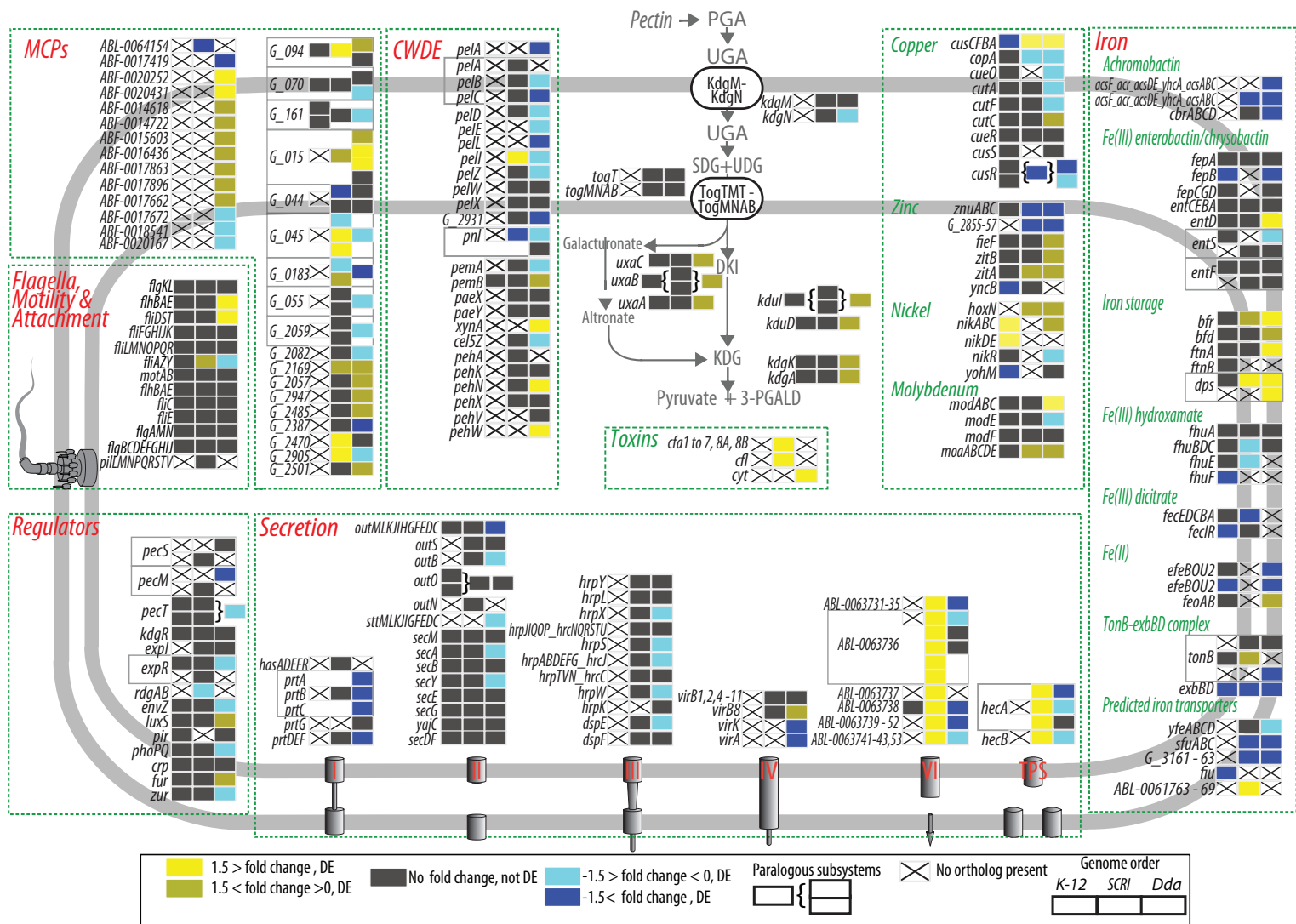

Supplement: Additional file 11 — Normalized signals, fold changes and significance for features in E. coli K12-MG1655. [file 1471-2164-13-110-S11.PDF]
